# Imaging assay to probe the role of telomere length shortening on telomere-gene interactions in single cells

**DOI:** 10.1007/s00412-020-00747-4

**Published:** 2021-02-08

**Authors:** Ning Zhang, Yanhui Li, Tsung-Po Lai, Jerry W. Shay, Gaudenz Danuser

**Affiliations:** 1 Lyda Hill Department of Bioinformatics, UT Southwestern Medical Center, Dallas, TX USA; 2 Department of Cell Biology, UT Southwestern Medical Center, Dallas, TX USA; 3 Center of Human Development and Aging, Rutgers New Jersey Medical School, The State University of New Jersey, Newark, NJ USA

**Keywords:** Telomere, Telomere position effect over long distance, Image analysis, Fluorescense In Situ Hybridization

## Abstract

Telomeres are repetitive non-coding nucleotide sequences (TTAGGGn) capping the ends of chromosomes. Progressive telomere shortening with increasing age has been associated with shifts in gene expression through models such as the telomere position effect (TPE), which suggests reduced interference of the telomere with transcriptional activity of increasingly more distant genes. A modification of the TPE model, referred to as Telomere Position Effects over Long Distance (TPE-OLD), explains why some genes 1–10 MB from a telomere are still affected by TPE, but genes closer to the telomere are not. Here, we describe an imaging approach to systematically examine the occurrence of TPE-OLD at the single cell level. Compared to existing methods, the pipeline allows rapid analysis of hundreds to thousands of cells, which is necessary to establish TPE-OLD as an acceptable mechanism of gene expression regulation. We examined two human genes, ISG15 and TERT, for which TPE-OLD has been described before. For both genes, we found less interaction with the telomere on the same chromosome in old cells compared to young cells; and experimentally elongated telomeres in old cells rescued the level of telomere interaction for both genes. However, the dependency of the interactions on the age progression from young to old cells varied. One model for the differences between ISG15 and TERT may relate to the markedly distinct interstitial telomeric sequence arrangement in the two genes. Overall, this provides a strong rationale for the role of telomere length shortening in the regulation of gene expression.

**Supplementary Information:**

The online version contains supplementary material available at 10.1007/s00412-020-00747-4.

## Introduction

Telomeres are repetitive nucleotide sequences (TTAGGGn) capping the end of chromosomes. The length of telomeres becomes progressively shorter after each cell division (Harley et al. 1990), which appears to serve as a clock or replicometer of human cellular lifespan. Telomeres prevent chromosome ends from fusion, degradation, and being recognized as double-strand DNA breaks (O'Sullivan and Karlseder 2010; Webb et al. 2013). Cells undergo replicative senescence when a single or perhaps a few telomeres become very short and unprotected, which results in DNA damage at telomeres (Zou et al. 2004). Importantly, previous work has shown that entry into cellular senescence and chromosome instability are dictated by the shortest telomere length, not the average telomere length (Hemann et al. 2001; Zou et al. 2004).

Germline cells and some highly proliferative stem-like cells can transiently express the ribonucleoprotein enzyme complex, telomerase, that can partially maintain telomere lengths (Wright et al. 1996). In contrast, the vast majority of adult somatic cells do not express telomerase. Thus, cells lose a small amount of telomeric sequences after each cell division due to incomplete DNA lagging strand synthesis also known as the end replication problem (Olovnikov 1971; Watson 1972). In addition, other factors such as oxidative stress may contribute to more rapid telomere erosion (Reichert and Stier 2017; von Zglinicki 2002). With progressive telomere shortening, cells will gradually reach a senescence checkpoint (Wright and Shay 1992b). Premalignant cells can bypass this checkpoint by acquiring p53 or pRB/p16 mutations to keep dividing (extended lifespan) until reaching a crisis checkpoint (Wright et al. 1989), at which step almost all cells will die. Only very few rare cells are capable of acquiring a method to maintain their telomere length in order to continue to divide (Shay and Wright 1989). While telomerase is almost universally activated at this stage, other mechanisms such as the alternative lengthening of telomeres (ALT) DNA recombination pathway have also been identified (Cesare and Reddel 2010). Once a telomere maintenance mechanism is achieved, cells reach a new steady state with unlimited potential to divide and this enables additional genetic and epigenetic changes leading to cancer development (Mathon and Lloyd 2001; Shay 2014).

**Fig. 1:**
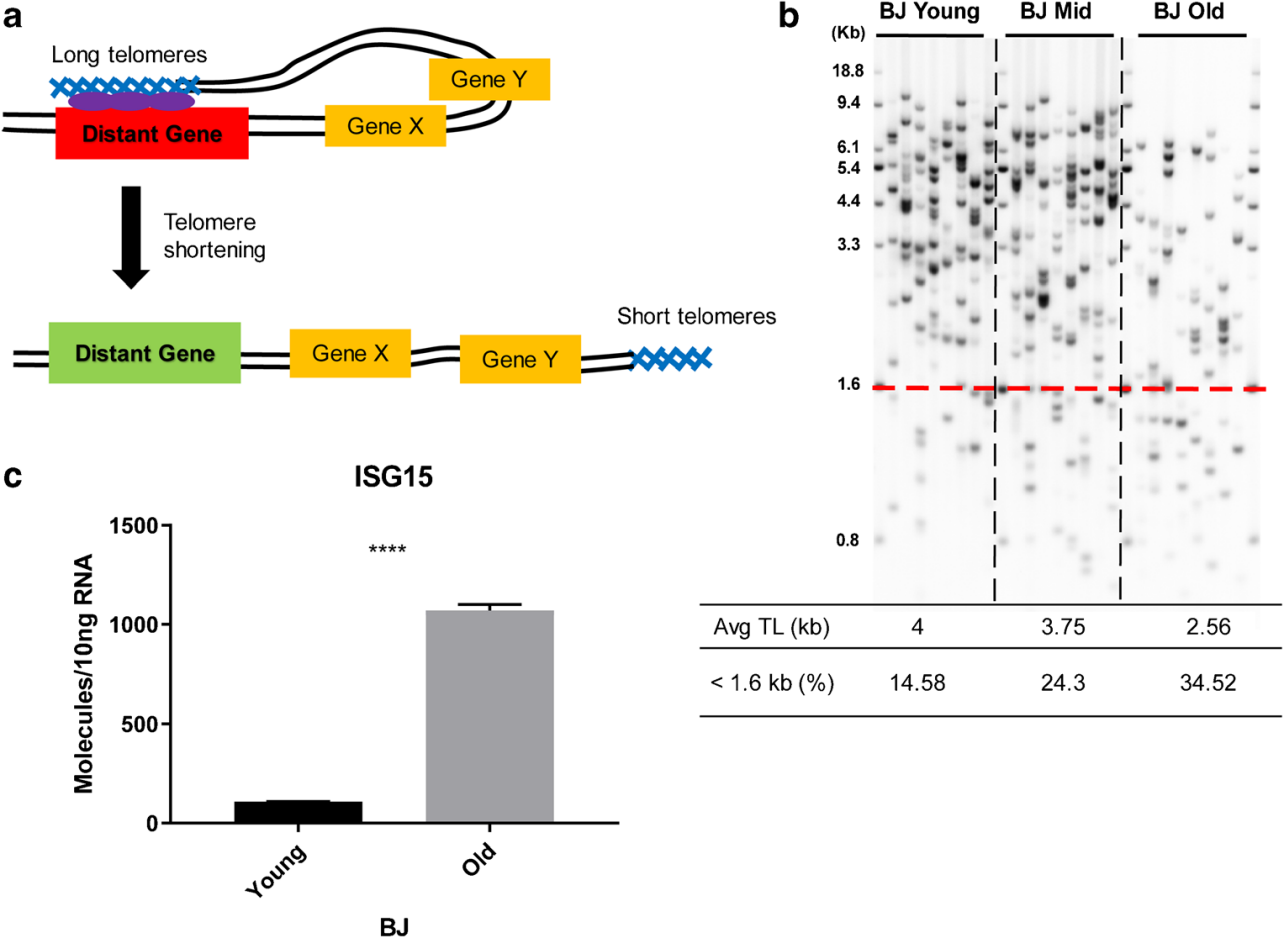
Working model of the TPE-OLD effect and of the gene expression level change in young/old cells. **a** Current model of telomere position effect over long distance (TPE-OLD). Long telomeres can form a loop and therefore interact with target genes over long distance. Red color indicates a repressed state, while green color indicates an activated state. Other genes (yellow color) sitting in between are not affected. Previous evidences support the assumption that the interaction might be mediated by the shelterin protein complex (purple color) and becomes weak as telomeres get short. **b** Telomere length distribution revealed by Telomere Shortest Length Assay (TeSLA) for human fibroblasts (BJ cells) with different population doublings (PD). Old cells tend to have smaller average telomere length and more short telomeres. Quantification of Southern blot bands was accomplished using the software described in (Lai et al. 2017). **c** ISG15 is a TPE-OLD candidate gene. The RNA expression level in young BJ cells is repressed (~ 10-fold lower than in old cells; *p* value < 0.0001; *n* = 3 experiments; error bars indicate standard deviation)

Studies have shown that the gradual decrease in telomere lengths can also regulate gene expression. Originally discovered in yeast, it was named telomere position effect (TPE) (Gottschling et al. 1990; Sandell and Zakian 1992; Stavenhagen and Zakian 1998; Wright and Shay 1992a), indicating that expression of genes adjacent to telomeres can be repressed. In mammalian cells, insertion of a luciferase reporter into the genome at short vs long distances from telomeres showed significant expression variation (Baur et al. 2001) with reduced luciferase expression when adjacent to a telomere but not when inserted far distances from a telomere. Interferon-stimulating gene 15 (ISG15) was the first endogenous mammalian gene reported as regulated by telomere length (Lou et al. 2009). It has low expression when telomeres are long, and gradually higher expression as telomeres get progressively shorter. The coupling between telomere length and gene expression for ISG15 was later reproduced in human fibroblasts and myoblasts (Robin et al. 2014; Stadler et al. 2013). In these studies, re-elongation of telomeres in older cells with short telomeres by expression of telomerase reversed the expression of ISG15. Curiously, telomere length-dependent expression was not observed for genes located between the telomere and specific target genes (e.g., ISG15 which is approximately 1 MB from the telomere). This modified form of TPE was termed Telomere Position Effect over Long Distance (TPE-OLD) or telomere looping (Kim et al. 2016; Mukherjee et al. 2018; Wood et al. 2014, 2015). The hypothetical model for TPE-OLD is that telomeres can loop back and interact with target genes with the help of shelterin protein complex (de Lange 2005; Kim et al. 2016). Short telomeres in old cells are unable to maintain such interactions and dissociate more frequently from the target genes, which leads to changes in gene expression (Fig. 1a). Therefore, TPE-OLD may explain why some genes relatively far away (up to 10 MB) from a telomere are regulated by telomere length shortening, yet other genes closer to the telomere are not. It further provides knowledge about how progressive telomere length shortening can affect aging-associated diseases before the DNA damage signal is triggered (Holohan et al. 2016; Li et al. 2019; Robin et al. 2015; Stadler et al. 2013).

Previous work in our lab identified that the expression of the human TERT (TElomerase Reverse Transcriptase) gene (Kim et al. 2016) is also related to telomere length. Compared to ISG15, the situation is complicated by the multiple alternative splicing isoforms of hTERT. Although there is a change of mRNA copy number for fragments between 5’UTR and Exon1, there is no RT domain (exons 5–10) expression observed in most normal somatic cells. That result is consistent with the observation that the vast majority of somatic cells do not have telomerase activity, but 85–90% of cancer cells have turned on telomerase to maintain the short telomere length. Furthermore, followed by the knockdown of p21 (mimicking a bypass of the cell senescence environment), the total hTERT mRNA in old cells was increased (Kim et al. 2016). This suggested that old cells prepare for TERT activation because p21 knockdown in young cells does not affect hTERT total mRNA. One way to prepare for TERT activation is to alter the interactions that repress interstitial telomeric sequences (ITS) activity, as discussed in this report.

To further investigate TPE-OLD genes like ISG15 and hTERT, and to understand the underlying mechanism, we need assays to probe gene expression variation in aging cell populations with a systematic and unbiased identification of interactions between target gene candidates and associated telomeres. It thus becomes important to develop higher throughput methods that can confirm the regulatory role of telomere length shortening on gene expression. Sequencing-based assays like chromosome conformation capture (3C) and Hi-C (de Wit and de Laat 2012; Denker and de Laat 2016; Lieberman-Aiden et al. 2009) have been widely applied on research of genome organization with large cell numbers. One previous study showed that Hi-C could capture potential long-range interactions on certain chromosomes (Robin et al. 2014). However, these assays require millions of cells and lack the distinction of gene-telomere interaction in individual cells.

In the present study, we applied a 3D-FISH imaging approach to quantify distances between the loci of a TPE-OLD gene and a unique subtelomeric region on the same chromosome. We observed that for ISG15 and TERT, the loci of fluorescent probe pairs have increased mutual distances (separation) when telomeres become short, indicating that telomere length shortening may regulate gene expression via mutual interactions in 3D. We also found that these distances were reversed in old cells whose telomeres were experimentally elongated by ectopic introduction of hTERT (catalytic rate limiting component of telomerase). We observed that at the population level, distance increases between TPE-OLD genes and telomeres are fairly rare events. Therefore, we implemented an automated pipeline, relying on 3D epifluorescence imaging, to acquire robust statistics of distance distributions. This allowed us to scrutinize the regulatory roles of progressive telomere shortening on gene expression levels. We observed that the loci of the ISG15 and TERT genes both have more interactions with their corresponding telomere on the same chromosome in young cells compared to old cells. With telomere length shortening, the telomere-associated interactions are gradually decreasing (like a rheostat) with protein levels increasing for ISG15. In contrast, the TERT progression appears to be more similar to an off/on switch.

The new results of this study and the technologies developed provide an easier platform for future work to systematically probe the significance of TPE-OLD as a mechanism regulating gene expression in normal human aging. The image analysis pipeline also provides automated and time-saving solutions on quantifying genome interaction in 3D.

## Methods

### Droplet digital PCR (ddPCR)

ddPCR was performed on samples using previously described protocols (Kim et al. 2016; O'Hara et al. 2019). mRNA was extracted from cell pellets using RNeasy plus mini kit (Qiagen) and reverse-transcribed using cDNA synthesis kit (Bio-Rad). Each 20 μL ddPCR reaction contained a final concentration of 1× EvaGreen ddPCR Supermix (Bio-Rad), 100 nM primers, and 10 ng cDNA sample. After PCR, fluorescent intensity of each oil droplet was measured using QX100 droplet reader (Bio-Rad). The threshold for positive droplets could be automatically or manually determined based on the baseline fluorescence level. The final software output generated quantitative results of template molecule concentration in 10 ng RNA.

### Telomere shortest length assay (TeSLA)

Genomic DNA was extracted from cell pellets using Gentra Puregene DNA Extraction Kit (Qiagen). TeSLA was performed as previously described (Lai et al. 2017). DNA (50 ng) was first ligated with TeSLA-T 1–6, followed by digestion using four restriction enzymes (*Bfa*I*/Cvi*AII*/Mse*I*/Nde*I, New England Biolabs). After dephosphorylation with rSAP (New England Biolabs), digested DNA was ligated with double-stranded adapters to ensure the amplification of telomeric DNA by PCR (94 °C for 2 min followed by 26 cycles at 94 °C for 15 s, 60 °C for 30 s, and 72 °C for 15 min).

PCR products were separated on a 0.85% agarose gel at 2 V/cm for 16 h. The DNA bands were first transferred to a Hybond-N+ membrane (GE) and then fixed by UV crosslinking. The membrane was then hybridized with DIG-labeled telomere probe at 42 °C overnight, followed by washing with buffer 1 (2× saline-sodium citrate (SSC), 0.1% sodium dodecyl sulfate (SDS)) at RT for 15 min, washing with buffer 2 (0.5× SSC, and 0.1% SDS) at 60 °C for 15 min twice, and washing with buffer 3 (1× maleic acid buffer with 0.3% Tween-20) for 5 min. The membrane was then incubated with 1× DIG blocking solution at RT for 30 min, followed by incubation of anti-DIG antibody (Roche) at RT for 30 min in 1× blocking solution (1 to 10,000 dilution). After washing with DIG buffer twice, telomere signals on membrane were detected by incubating in dark with CDP-star (Roche) for 5 min. Telomere band image was taken by G:box. The average telomere length and percentage of short telomeres (below 1.6 kb) were quantified using TeSLA Quant software.

### Cell culture and fixation

Primary human fibroblasts (BJ cells) were cultured in Medium X (DMEM: Media199 = 4:1, Hyclone) containing 10% cosmic calf serum (Hyclone) at 37 °C. Cells with different population doublings (PD) were passaged using 0.05% Trypsin-EDTA (1X, Gibco) and 20–50 K cells were seeded in each chamber of the 4-chamber glass slide (LAB-TEK).

Slide chambers were manually removed after overnight incubation. Cells on slides were fixed with 4% paraformaldehyde (PFA), followed by cytoplasm washout with 2% PFA/0.5% Triton X-100. Then, cells were permeabilized with 0.5% Triton X-100 in PBS and incubated in 20% glycerol/PBS for at least 20 min. 3X liquid nitrogen freezing-thawing cycles were then performed on slides for further permeabilization. Slides were incubated again in 20% glycerol/PBS at RT for 1 min after each cycle, followed by 5 min wash in 1X PBS and 30 min incubation in 0.1 M HCl. Finally, slides were preserved at 4 °C in 50% formamide (pH 7)/2X SSC in a Coplin jar shielded from light.

### Hybridization of probes

Slides with cells were stained with hybridization probes ISG15-FITC, TERT-FITC (Empire Genomics), 1pter and 5pter subtelomere specific probes, Texas Red (Cytocell) following the vendor’s protocol. The hybridization lasted overnight at 37 °C in a light-tight humidified chamber. The next day, slides were sequentially washed with 0.2X SSC, 2X SSC, and 1X PBS, stained in 1 μg/ml DAPI solution and mounted with Fluoromount-G (ThermoFisher).

### Image acquisition

Images were acquired in 3D using a Nikon Ti-Eclipse widefield microscope equipped with 60X/NA = 1.4 oil lens, a CMOS camera, and filters for DAPI, FITC and TRITC. The alignment of microscope channel was qualitatively checked using TetraSpeck Microspheres (Invitrogen). For the fine correction of residual chromatic aberration, we implemented a compensation schema at the level of the distance calculation (see Image Analysis, below). For each slide, multiple, non-overlapping fields of view for acquisition of 3D stacks were defined on the positions of a square grid with an interval of 0.5 mm. After the stage was moved to the stack position, stacks were acquired over a range of ± 10 μm above and below the set focal plane with a voxel size of 108 nm × 108 nm × 293 nm.

### Image analysis

A 3D data analysis pipeline was written in MATLAB. It offers user-friendly step-by-step analysis. The pipeline comprises five modules: image preprocessing, nuclei segmentation, spots detection, spots pairing, and distance analysis.

#### Image preprocessing

Multiple 3D stack images were sequentially read and analyzed using a custom-written image processing pipeline. After reading a stack, the voxel intensities in each channel were normalized to the min/max range of [0, 1].

#### Nuclei mask segmentation in 3D

Nuclei masks were segmented from the DAPI channel. The pre-processed DAPI channel was compressed to 2D by maximum intensity projection and then convolved with a Gaussian filter with σ_X/Y_ = 3.75 μm, matching approximately the size of a fibroblast nucleus. Local intensity maxima in the filtered image were marked as the centers of 45 × 45 μm^2^ squares. Bounding boxes of 45 × 45 μm^2^ × stack height were defined about these local maxima. Only boxes containing one maximum were further processed. The original cell seeding density was calibrated to minimize the overlap bounding boxes.

The actual nucleus volume in every bounding box was segmented slice by slice in 2D and then assembled in 3D. The 2D segmentation integrated the information from multi-scale filtering and thresholding, followed by majority voting to determine whether pixels fell inside or outside the nucleus volume. Given the fact that top/bottom slices are blurred and should contain less nuclei voxel, global thresholding was performed to refine the 3D mask so that any voxels with intensities less than 0.6 × global threshold were marked as background. Nuclei with extreme volume sizes or touching the image boundary were removed automatically. On average, a single field of view contained ~ 10 valid nuclei for further processing.

#### Spot detection

Nuclei masks extracted from the DAPI channel were then applied to the fluorescent channels to detect in each nucleus independently FISH probes as diffraction-limited spots using a previously described pipeline (Aguet et al. 2013; Roudot et al. 2017). These algorithms apply statistical testing for the selection spot signals deemed as significantly brighter than the background. *P* values ranged from 0.05 to 0.1, intentionally set to suppress false negatives at the risk of a higher false positive rate. The breakdown of spot detection into a nucleus-by-nucleus protocol was essential to account for the vast difference in fluorescent background between nuclei.

#### Spot pairing

Corresponding pairs of FITC and TRITC spots were identified by solving linear assignment problem in bipartite graph. In brief, the graph was computed by considering all possible spot pairs i, j in the FITC and TRITC channels, respectively, with a 3D distance less than 5 μm. The spot candidate intensities (I_i_ and I_j_) were also recorded to calculate a pairing score ${S}_{ij}={d}_{ij}/\sqrt{I_i{I}_j}$., i.e., spot pairs with high brightness and short distance had the lowest scores. We then applied a modified Hungarian algorithm (Kuhn 1955) to identify among all possible pairs in the graph the two mutually exclusive pairs among the pair assignments with overall smallest score. The scores of the two selected pairs tested against the scores of all other pair assignments. We required that their scores be 50% smaller than the following spot pair score (Fig. S2). Nuclei for which this requirement was not fulfilled were eliminated from the data set.

#### Correction of chromatic shifts

Before compiling the selected spot pairs into distance distributions reflecting the telomere-target gene interaction under a particular experimental condition, we eliminated distance bias due to FITC-TRITC channel misalignment (chromatic aberration). To accomplish this for one particular experiment, we computed the mean 3D displacement vector from the FITC to the TRITC spot and subtracted it from the individual displacements, i.e., the corrected displacement vector distribution has a mean value of [0, 0, 0]. Distance distributions for statistical analysis were then computed based on these corrected vectors. The underlying assumption of this correction protocol is that the vast majority of displacement vectors represent short, random distances between interacting subtelomeric and target gene sequences with no preferred spatial directionality.

### Analysis of interstitial telomeric sequences (ITS)

ITS analysis was performed using IGV software (Thorvaldsdottir et al. 2013). Specific motifs of TTAGGG and the complementary sequence CCCTAA were searched using reference genome hg19. We defined the search region on chromosomes 1 and 5, respectively, to cover the entire gene with flanking sequences of 19 kb for ISG15 and 56 kb for TERT. The searching region was visualized in the software output panel with indicated positions of RefSeq Genes and target motifs. We further plot red arrows to highlight the ITS positions.

## Results

### Gene expression level changes with telomere length shortening

The relation between telomere length shortening and gene expression regulation under the TPE-OLD mechanism is thought to rely on the interaction between telomere and gene locus, which is mediated by the shelterin protein (Kim et al. 2016; Kim and Shay 2018; Robin et al. 2014, 2015; Stadler et al. 2013) (Fig. 1a). To measure how the interaction is affected by telomere length shortening in individual cells, we first prepared human fibroblasts (BJ cells) with different ranges of population doubling (PD). Young cells (PD 14–32) were expected to have the longest telomeres, and mid-age cells (PD 33–50) and old cells (PD > 50) were expected to have gradually shorter telomeres. To validate this expectation, we performed a Telomere Shortest Length Assay (TeSLA) on all three cell populations. TeSLA allowed us to measure the telomere lengths in a mixed population of cells with a sensitivity for short telomeres below 1.0 kb (Lai et al. 2017). As expected, the average telomere length became shorter when cells grew older, and the percentage of the shortest telomeres (< 1.6 kb) increased (Fig. 1b). We then tested the expression level of a previously identified TPE-OLD gene, ISG15, using droplet digital PCR (ddPCR). Indeed, in young cells, the mRNA copy number was 10-fold lower than in old cells (Fig. 1c) and correlated with an increase in ISG15 protein levels [22].

### Determining telomere-gene interactions using single-cell imaging

**Fig. 2:**
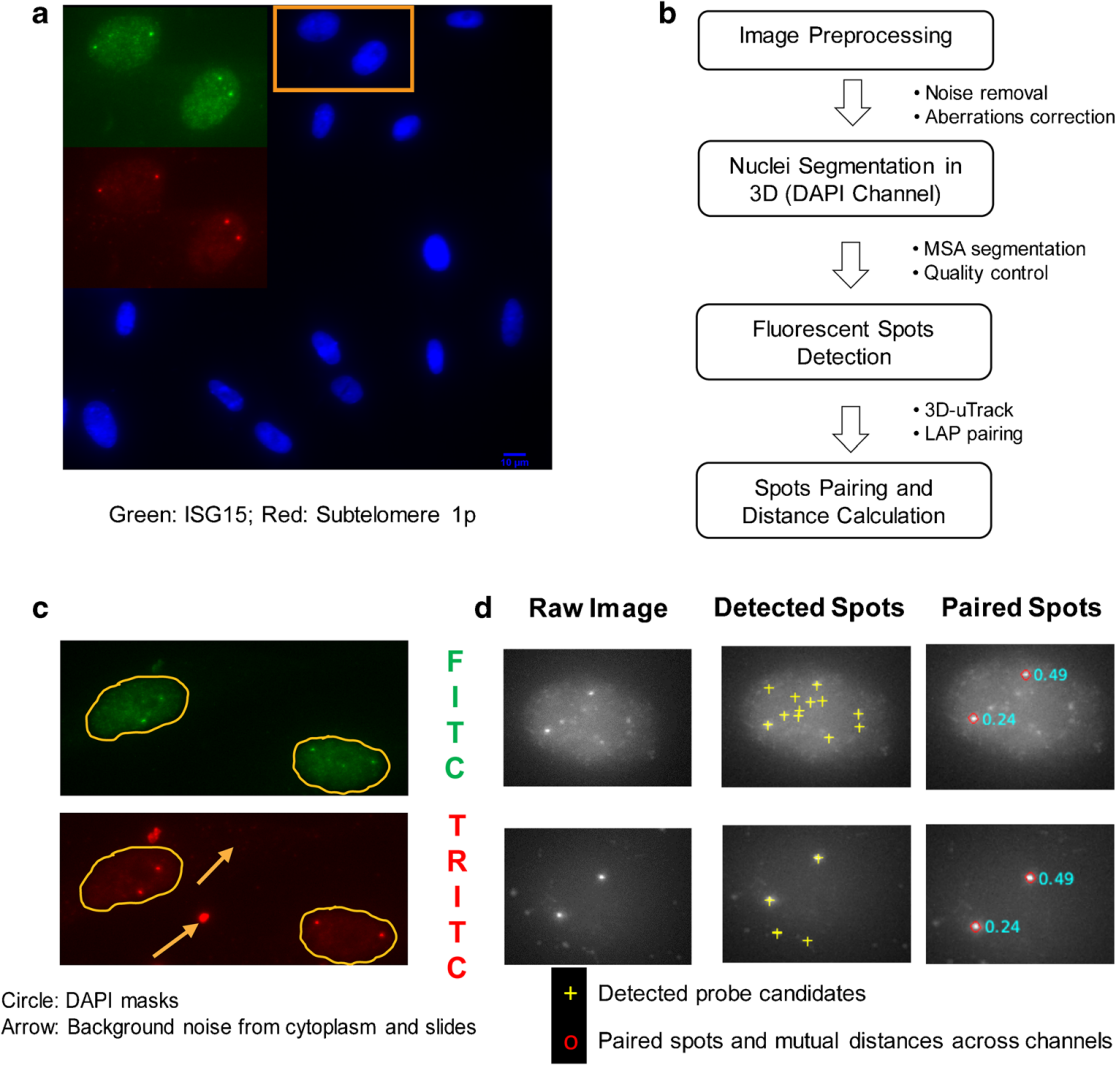
Visualization and analysis of the relative position of chromosome-specific subtelomere sequence and target gene locus by FISH. **a** Maximum intensity projection (MIP) of a 3D sample image showing nuclei in DAPI channel. Inset: Zoomed version of MIP of FITC (green; ISG15 loci) and TRITC (red; specific subtelomeric region near chromosome 1p) channels associated with the nuclei indicated in the overview. The selected nuclei show a prototypical scenario with two clearly discernible spots per channel, which co-localize between channels. **b** Overview of image analysis pipeline. **c** MIP of FITC and TRITC channels indicating representative scenarios of FISH probe clutter (arrows) outside the nuclear perimeter as well as ambiguous spot signals inside the nucleus. **d** MIPs of FITC and TRITC channels indicating representative scenarios of FISH probe signals (left). Both channels contain more than two discernible spots, all of which are correctly detected as probe candidates based on the statistical significance of foreground to background contrast (middle, yellow crosses). Based on a pairing algorithm and selection of the overall two brightest pairs, FISH probes tagging bona fide subtelomeric and target gene sequence are identified (right, red circles). Cyan numbers in the right column indicate the distance between corresponding probes in micrometers

We next developed an automated image acquisition and analysis pipeline to determine the level of interaction between genes and subtelomeric regions on the same chromosome by single-cell screening in 3D. To visualize the interaction between telomeres and TPE-OLD genes, we labeled the locus of a chromosome-specific, subtelomeric sequence and the locus of a target gene of interest on the same chromosome using FITC- and TRITC-tagged FISH probes, respectively. We also labeled the overall nuclear volume using a DAPI stain. After fixation and labeling, 3D image stacks were acquired to measure the distances of telomere-gene pairs. Figure 2a shows a typical field of view in maximum intensity projection (MIP), with the DAPI channel outlining the nuclei and the zoom-in windows presenting the FITC and TRITC channels for two neighboring nuclei. Both FISH-probe channels indicate four clearly discernible bright spots in the same location, suggesting co-localization of the subtelomeric probe and the gene of interest, and thus presumptive interaction, for both chromosomes in each nucleus. More examples are provided in Fig. S1.

We expected that in a population of cells, the presence or absence of telomere-gene pairing would be heterogeneous, both because of variability in the DNA sequence interaction per se and variability in telomere length at the individual cell level (Fig. 1b). Although previous studies have used manual analyses of small cell populations to demonstrate the shift in telomere-gene interactions between cells with long and short telomeres (Robin et al. 2014), it was unclear for a general case how many cells would be necessary to detect shifts in TPE-OLD regulation between two conditions. To investigate this question using an unbiased data set, we developed a protocol based on a motorized microscope stage to acquire 3D stacks of hundreds to thousands of cells on a single slide. For each slide, we typically sampled 50–80 randomly placed fields of views, each containing ~ 15 cells, on average. We employed widefield epi-fluorescence imaging because of the higher detection sensitivity and the ten-fold faster image acquisition rate compared to confocal microscopy.

We then implemented a fully automated image processing pipeline, delineated in Fig. 2b, to systematically measure the relation between the FISH markers of the subtelomeric region and TPE-OLD gene of interest in every nucleus. After image pre-processing steps, such as pixel intensity normalization and background removal, we segmented each nucleus from the DAPI channel (Fig. 2c, see the “Methods” section for details) and then applied a sub-pipeline for FISH marker detection for each nucleus separately. This eliminated contaminating signals in the space between nuclei (Fig. 2c, yellow arrows) and allowed an adaptive tuning of image filters and thresholds for FISH probe identification between nuclei with very different fluorescent backgrounds.

Specifically, in each nuclear volume, we first detected spots in 3D in both fluorescent channels, adapting a 3D version of the previously published algorithm by Aguet et al. (Aguet et al. 2013). Statistical spot selection was performed at a *p* value of 0.1, which tends to err on the side of false positive candidates. This ensured that the initial spot sets included the signals of all FISH markers with high confidence.

Next, we sought to identify spot pairs between the FITC and TRITC channels that would represent with high likelihood the interaction between a telomere and TPE-OLD gene marker. We made the assumption that both markers produce a relatively bright spot and that the proximity in 3D of corresponding markers, on average, is much greater than the proximity of randomly paired spots, even in the case where the DNA sequences of telomere and TPE-OLD gene do not interact. To capture this model, we computed a pairing score matrix between FITC and TRITC channel spots. Scores were low for bright and proximal spots, whereas scores were high for dim and distant spots. Based on this score matrix, we assigned spot pairs by solving the linear assignment problem (LAP) (Jaqaman et al. 2008; Jonker and Volgenant 1987), which identified among all pairing configurations the one with the overall smallest sum of scores. Due to the detection of an unequal number of spots in both channels, our LAP implementation accounted for the case in which not every spot in one channel is paired to a spot in the other channel. Finally, our algorithm verified that the pairing scores of the two lowest score assignments were significantly less than the scores of any other pairing (Fig. S2). Only nuclei fulfilling this condition were accepted as containing valid states of telomere and TPE-OLD gene interactions. Figure 2d provides an example of spot detection and pairing. Note that in this particular case, the FITC channel contains several nearly identically bright spots; however, the selection of the two relevant FISH markers is unambiguous when considering the detections in the TRITC channel. Figure S2 displays additional examples of high- and unacceptably low-confidence pairings.

### The increased separation between the gene of interest and subtelomere along with cell replicative aging

**Fig. 3:**
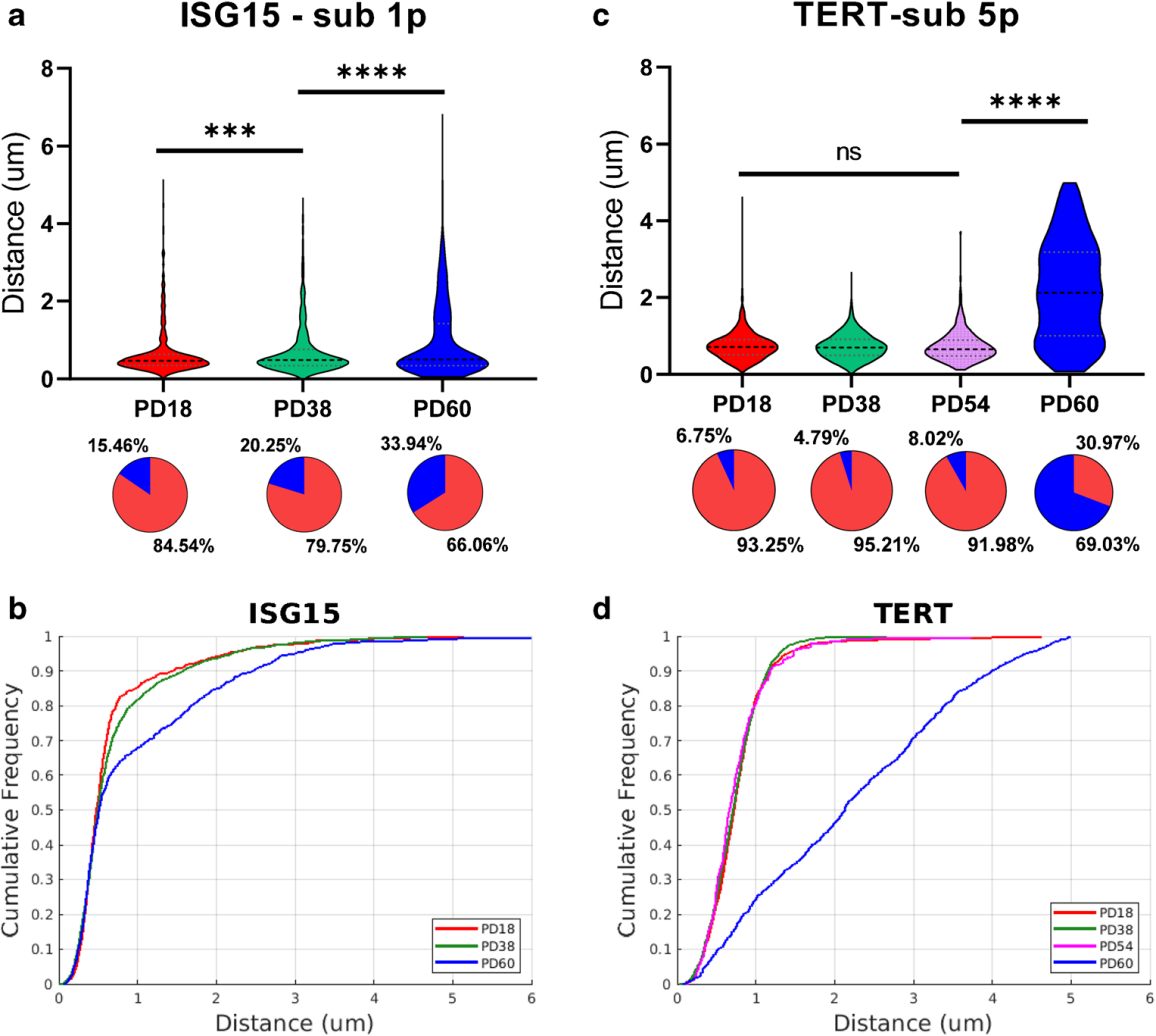
TPE-OLD genes interact more with long telomeres. **a** Violin plots of distances between ISG15 and subtelomere 1p for BJ fibroblasts with PD18 (*n* = 414 cells), PD38 (*n* = 1079 cells), and PD60 (*n* = 416 cells). 25%, 50%, and 75% quantile lines are plotted. Kolmogorov–Smirnov (KS) test was applied to determine the significance of differences between the distributions under different conditions. The significant *p* values were observed between any two distance distributions (*p* < 0.001). The distance distributions were segmented using 0.9 μm cutoff to separate the interacting main lobe vs. non-interacting long tail, as illustrated by the pie charts. **b** Empirical cumulative distribution function (CDF) plots of distances between ISG15 and subtelomere 1p for BJ fibroblasts with PD18, PD38, and PD60. Young cells have more short-distance pairs, suggesting closer interaction between the telomere and ISG15. **c** Violin plots of mutual distances between TERT and subtelomere 5p for BJ fibroblasts with PD18 (*n* = 704 cells), PD38 (*n* = 835 cells), PD54 (*n* = 162 cells), and PD60 (*n* = 536 cells). KS test was applied to check if two distributions are significantly different from each other. The significant *p* value was only observed between PD60 cells and other groups (*p* < 0.0001). The distance distributions were segmented using 1.3 μm cutoff to separate the interacting main lobe vs. non-interacting long tail, as illustrated by the pie charts. **d** CDF plots of paired distances between TERT and subtelomere 5p for BJ fibroblasts with PD18, PD38, PD54, and PD60. Young cells have more adjacent pairs, which indicates closer interaction between the telomere and TERT

Equipped with this imaging pipeline, we first investigated the gradual separation of a well-established TPE-OLD gene, ISG15 (Lou et al. 2009), from the corresponding telomere on chromosome 1p as cells get older. Regardless of cell age, represented by the population doubling (PD), the vast majority of parings had a 3D distance of less than 500 nm, i.e., the spots in FITC- and TRITC-channel fell within the same point spread function and thus appear visually co-localized (Fig. 3a). With increasing age, an increasing sub-population of nuclei with distances of between 500 nm and 3 μm is detected suggesting that a larger number of telomeres dissociated from the ISG15 locus. Importantly, at all ages, these longer distance pairs describe the exception to the rule. This implies that the expression shifts of TPE-OLD genes (Fig. 1c) are driven by only a small sub-population of cells, and bulk measurements of DNA-DNA interactions, like 3C and Hi-C, are relatively insensitive in detecting TPE-OLD. Even with a single-cell assay as described here, TPE-OLD can only be confirmed based on a statistical sample large enough to capture a representative outlier population. To visualize the shift in the outlier population, we present the cumulative distributions (Fig. 3b). In this representation, it becomes obvious that interactions between ISG15 and the subtelomere is decreasing as the PD increases. The significance of these shifts is quantified by the Kolmogorov-Smirnov test statistics (Massey 1951) (Fig. 3a). Rosin thresholding (Rosin 2001) was applied to segment the unimodal distance distribution for each PD into a sub-population of interacting gene-subtelomere pairs (main lobe) and a sub-population of non-interacting gene-subtelomere pairs (long tail), as illustrated in the pie charts. No significant difference was found in control experiments, where cell populations were compared between different days or between different glass slides on the same day (Fig. S3).

### Large cell number quantification is required for statistical robustness

According to our analysis thus far, detecting of TPE-OLD therefore relies on the assessment of the abundance of data outliers. This is a notoriously difficult task, strongly depending on a representative and geometrically unbiased sampling of the subtelomere-gene distances for a particular experimental condition.

Therefore, we next investigated how many cells should be imaged to confidently identify a TPE-OLD gene. Specifically, we determined by random sampling of increasingly larger cell subpopulations the number of cells required to reveal the distance distributions difference between age groups. For each subpopulation size, we bootstrapped 500 samples from the full cell populations of two age groups (younger and older cells) and then computed the percentage of samples yielding a significant difference between younger versus older cells. We set the condition for a high-confidence distinction between any two age groups as 98% or more of sample pairs be assessed as different. As illustrated in Fig. S4, at least 150 cells are needed to recapitulate the difference between distance distributions of young and old BJ cells for ISG15-associated telomere interactions. More cells are required to capture the distribution differences if the experimental conditions have a closer age gap.

### Identification of an age-controlled switch in the TPE-OLD-based repression of the TERT gene

TERT is the reverse transcriptase protein core component of the telomerase complex, which promotes elongation and partial maintenance of telomere length in stem cells and stable maintenance of telomere length in more than 90% of cancer cells (Shay 2016). A previous study suggested that TERT is another TPE-OLD gene in large long-lived mammals (Kim et al. 2016). We sought to confirm this in our “aging”-model of human cells. In contrast to the ISG15 gene, we observed that the distance distributions between TERT and associated telomere remained constant up to PD 54, with most mutual distances less than 1 μm (Fig. 3c). Beyond PD54, there was an abrupt change in the distribution, where a significant fraction (49% of PD 60 fibroblasts) fell outside the 99%-quantile (2.13 μm) of the distance distributions of PD54 and younger. To better visualize the switch-like shift, we present the cumulative distributions (Fig. 3d). We found that the distance distributions of PD 18, 38, and 54 did not significantly differ. Since TERT has no activity in most somatic cells (Wright et al. 1996), in previous work, we have proposed that TPE-OLD is among the primary mechanisms of repressed telomerase activation during human fetal development [26]. Our present data supports this notion with a direct experiment in a cell-level “aging” model and identifies a switch-like release of the repression beyond a critical replication count.

### TERT-immortalized cells observe more interaction between TPE-OLD genes and the subtelomeres in late passage normal cells

**Fig. 4:**
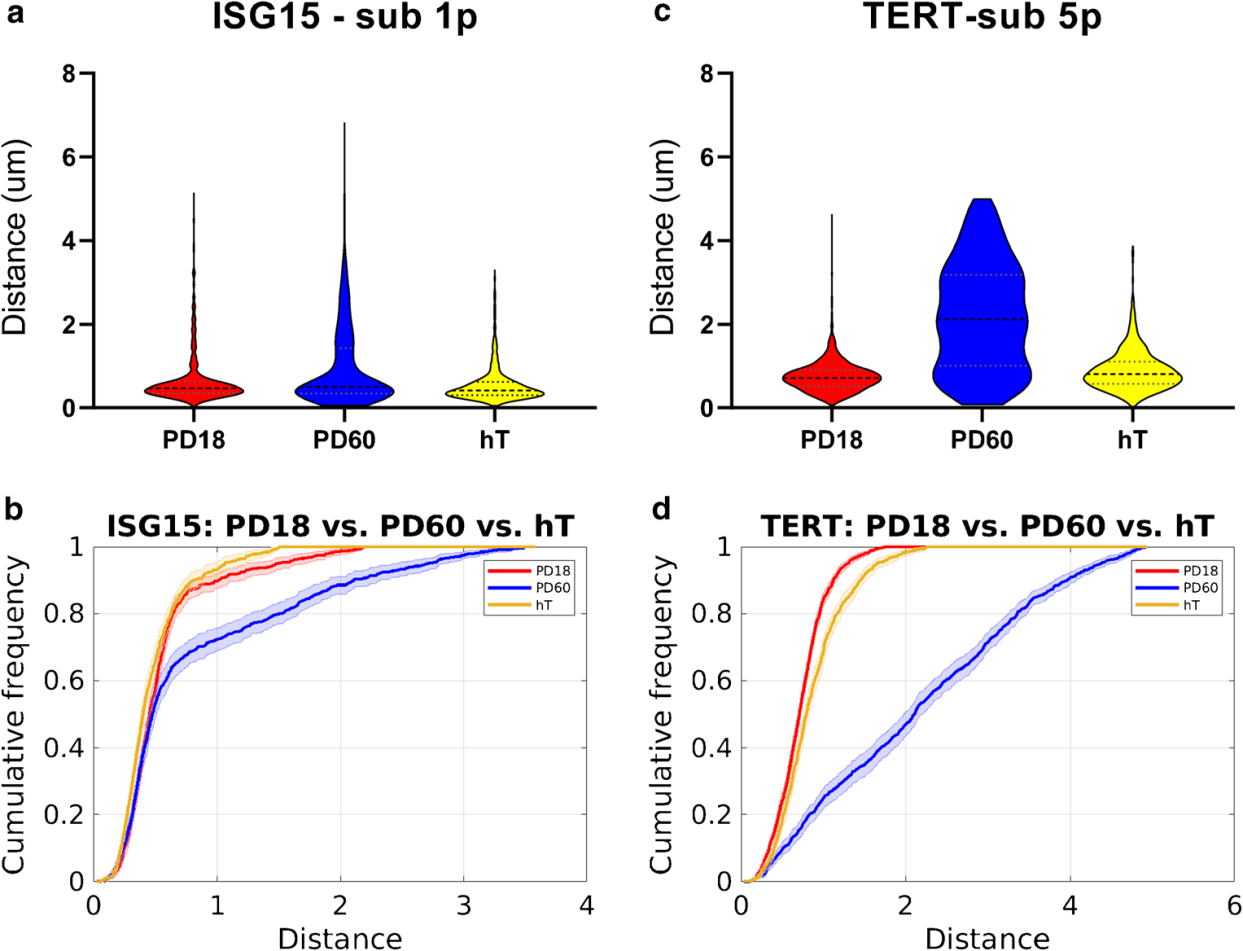
TERT immortalized cells reverse the telomere-gene interaction pattern. **a** Violin plots of distances between ISG15 and subtelomere 1p for BJ fibroblasts with PD18 (*n* = 414 cells), PD60 (*n* = 416 cells), and TERT immortalized (hT; *n* = 345 cells) cells. 25%, 50%, and 75% quantile lines are plotted. **b** Cumulative histograms of the same distances. **c** Violin plots of distances between TERT and subtelomere 5p for BJ fibroblasts with PD18 (*n* = 704 cells), PD60 (*n* = 536 cells), and TERT (*n* = 437 cells) immortalized cells. 25%, 50%, and 75% quantile lines are plotted. **d** Cumulative histograms of the same distances

Our observation of an increased dissociation of subtelomeres from both the ISG15 and TERT gene loci in old cells (PD60) led to the obvious hypothesis that the loss of interaction directly relates to the shortening of telomeres in an aging cell population, i.e., shorter telomeres have lower probability of interacting with a TPE-OLD gene. Testing this directly would require a concurrent assay of subtelomere-gene interaction and telomere length at the single cell level. Such an assay is currently not feasible. To nonetheless probe the effect of telomere length on the TPE-OLD genes, we tested BJ cells with hTERT reintroduced in late passage (Bodnar et al. 1998) and observed the distance distributions for the ISG15 and the TERT genes (Fig. 4). Confirmed by TeSLA, the telomere length of immortalized BJ cells is significantly re-elongated (Fig. S5). Intriguingly, for both genes, hTERT expression restored telomere length in old cells back to the distance distributions of PD18 cells, directly supporting the hypothesis that the TPE-OLD mechanism is controlled by telomere length.

### Interstitial telomeric sequences (ITS) discovered nearby the genes

**Fig. 5:**
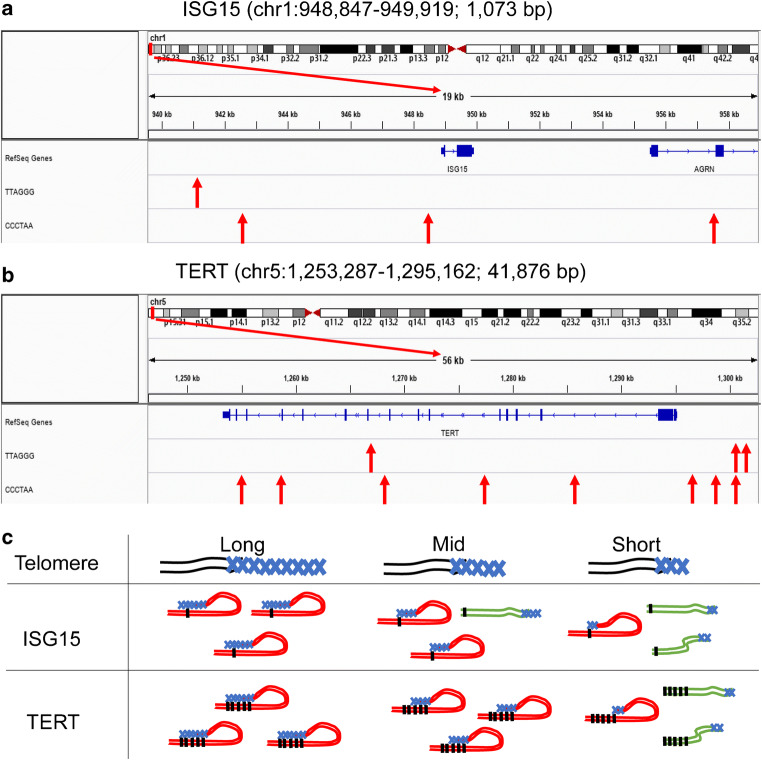
Model of telomere-gene interactions modulated by interstitial telomeric sequences. **a** Positions of interstitial telomeric sequence (ITS, TTAGGG) and complementary sequence (CCCTAA) indicated by red arrows within and flanking the ISG15 gene. ISG15 gene length and position are indicated. Total lengths of flanking regions, 19 kb. **b** Positions of (TTAGGG) and complementary sequence (CCCTAA) indicated by red arrows within and flanking the TERT gene. TERT gene length and position are indicated. Total lengths of flanking regions, 56 kb. **c** Illustrations of gene-telomere interaction change along with telomere length shortening for ISG15 and TERT. For ISG15, the interaction probability is proportional to the telomere length due to the limited number of ITS. Thus, gradual shortening leads to gradual loss of interaction. In contrast, the multiple ITSs within and flanking TERT may result in a binding cooperativity that causes the interaction probability to be insensitive to telomere shorting up to the point at which the telomere is too short to bridge multiple ITSs

An emerging area of interest is the role of telomere 3D looping with ITS (Simonet et al. 2011), i.e., TTAGGG, through interactions with telomere shelterin proteins (Wood et al. 2014). Telomeres form loops that interact with ITS at distal genomic regions. In the case of ISG15, the interactions likely occur via single ITS within 3 kb from the start codon (Fig. 5a), while the TERT gene includes many more ITS that provide potential interaction sites (Fig. 5b). Hence, in the ISG15 case, the interaction probability is directly proportional to the telomere length, and thus gradual shortening in a heterogeneous population of cells leads to gradual loss of TPE-OLD. In contrast, the multiple ITS in and surrounding the TERT gene may result in a binding cooperativity that causes the interaction to be insensitive to telomere shortening up to the point at which the telomere is too short to bridge multiple ITS (Fig. 5c). We also searched for multiple TTAGGG repeats on a broader range and found ~ 50 nucleotides ITS at the distance of about 100 kb downstream from TERT. This potentially explains the switch-like loss of telomere-gene interactions for TERT.

## Discussion

Telomere length shortens along with cell division and aging in all normal human somatic cells. It has been postulated that this shortening can affect the expression level of genes at distances up to 10 MB from the chromosome end, a mechanism referred to a TPE-OLD or telomere looping (Kim et al. 2016; Kim and Shay 2018; Lou et al. 2009; Robin et al. 2014, 2015; Stadler et al. 2013; Wood et al. 2015). To provide more insights of gene-telomere interactions, we developed an automated pipeline for measuring in hundreds of cells individually the proximity of a unique subtelomere sequence of a chromosome and a potential TPE-OLD regulated gene locus on the same chromosome. This amount of data is required to capture with sufficient confidence the consequences of intrinsically stochastic and convoluted processes that determine the proximity of subtelomere and target gene marker. The pipeline encompasses image preprocessing, nucleus segmentation, and fluorescent spot detection and pairing, from which 3D distance distributions are derived as proxies of telomere-gene interactions.

Although the distance distributions are defined on a continuous spectrum, the information gleaned from the distance distribution is essentially binary (illustrated by the supporting pie charts in Fig. 3). The gene and sub-telomere are either interacting, which yields a configuration with co-localized probes or non-interacting, which yields probe pairs separated by distances significantly above a threshold demarcating the main lobe of short distances between co-localized probes. Even in old cells, the vast majority of telomere-gene pairs is interacting. These interactions are likely quite strong and do not change with cell cycle dependent chromosomal rearrangements, such as those measured by Hi-C (Nagano et al. 2017). Once an interaction is released, the distance between the probes is arbitrary. It is now a product of many factors (chromatin entanglements, passive and probably active forces displacing the gene and telomere loci, nuclear volume, and perhaps cell cycle, etc.) These factors may also be at play as breakers of the telomere-gene interactions. However, we interpret our data to suggest that telomere sequence length is the strongest driver of the ratio between interacting and non-interacting gene-sub-telomere pairs. Regardless of the mechanism, the described assay permits the precise measurement of effects that govern telomere-gene interactions.

Since the vast majority of gene-sub-telomere distances were short, any further dissection of the TPE-OLD mechanism requires high-throughput measurements in order to capture the subtle interaction shifts between experimental conditions. Biologically, this implies that TPE-OLD defines a mechanism for stepwise activation of transcriptional activity and protein expression of a small set of select genes in individual cells.

Intriguingly, because of the unbiased acquisition of single-cell measurements, our data revealed two genes with differential long-term kinetics of gene-transcription changes with telomere shortening. While the ISG15 gene becomes gradually released from telomere interactions with progressive telomere shortening, the TERT gene displayed no significant interaction level change between young and middle-aged cells. The release of the TERT gene from telomeric interactions occurred only in the oldest population we could analyze. Given the fact that about 90% of cancer cells with short telomeres have telomerase activity (Jafri et al. 2016; Kim et al. 1994; Shay and Bacchetti 1997), there might be a mechanism that enhances telomerase activation specifically in old cells. A previous study (Kim et al. 2016) also found the enrichment of telomere-associated shelterin protein component TRF2 near the hTERT promoter via ChIP. Young cells tend to have more TRF2 interactions with TERT, which is consistent with the observation from our large-scale image data analysis.

In summary, our assay sets the foundation for a systematic validation and mechanistic analysis of candidate genes whose expression regulation may be co-regulated by TPE-OLD. In a previous study, we found that DNA methylation and histone modifications in the hTERT promoter region showed significant changes as cells developed shorter telomeres and that TRF2, a TTAGGG shelterin protein, may have important roles in these age-dependent genomic changes. These observations offer a model and a partial explanation for how age-dependent changes in the genome structure potentially affect the regulation of genes without initiating a DNA damage response from a critically shortened telomere. In conclusion, changes in telomere looping with increased age (and progressive telomere shortening) may be one mechanism of how cells time changes in physiology over decades. With the improved higher throughput single-cell imaging approach described here, it will now be possible to acquire more knowledge of TPE-OLD genes.

## Supplementary Information

Fig. S1 Maximum intensity projection (MIP) of representative examples with the DAPI channel outlining the nucleus boundary and FITC and TRITC channels showing the gene probe and the sub-telomere probe, respectively. (PNG 260 kb).

High resolution image (TIF 15486 kb).

Fig. S2 Quality control of spot pairing. (**a**) Examples of high-performance hybridization and low performance hybridization in spot pairing. Red circles indicate the positions of top 2 pairs in each nucleus. (**b**) Ranked pairing scores of selected nuclei. With high performance hybridization, the top two candidates have significantly lower score compared to the other scores. With low performance hybridization the scores of the two candidates are not clearly different from the other scores. (PNG 604 kb).

High resolution image (TIF 63431 kb).

Fig. S3 hTERT immortalized cells analyzed between (**a**) two different days or (**b**) on the same day, but on different glass slides. No significant difference was found between the distributions of these data groups (*p*-values of 0.1 and 0.08 for the day-to-day and same-day comparisons, respectively). (PNG 236 kb).

High resolution image (TIF 24439 kb).

Fig. S4 Large cell number quantification is required for statistical robustness. Large cell number is required for quantification. Designated number of cells were randomly selected from the PD18, PD38 and PD60 cell pools using bootstrap. Only with more than 150 cells can the difference between PD18 and PD60 be reproducibly observed. More cells are required if the two samples have closer PDs. (PNG 220 kb).

High resolution image (TIF 19503 kb).

Fig. S5 TeSLA of TERT immortalized BJ cells shows the re-elongated telomere length. Telomere length distribution revealed by Telomere Shortest Length Assay (TeSLA) for hTERT immortalized old human fibroblasts (BJ cells). Its telomere length was re-elongated due to the function of telomerase after cultivation. (PNG 148 kb).

High resolution image (TIF 24404 kb).

## Data Availability

Reagents, cells, and images are available upon request.
