# Supplementary figures and images for: Imaging assay to probe the role of telomere length shortening on telomere-gene interactions in single cells
Source: Chromosoma. 2021 Feb 8;130(1):61–73. doi: 10.1007/s00412-020-00747-4 (PMC7889534; doi:10.1007/s00412-020-00747-4)

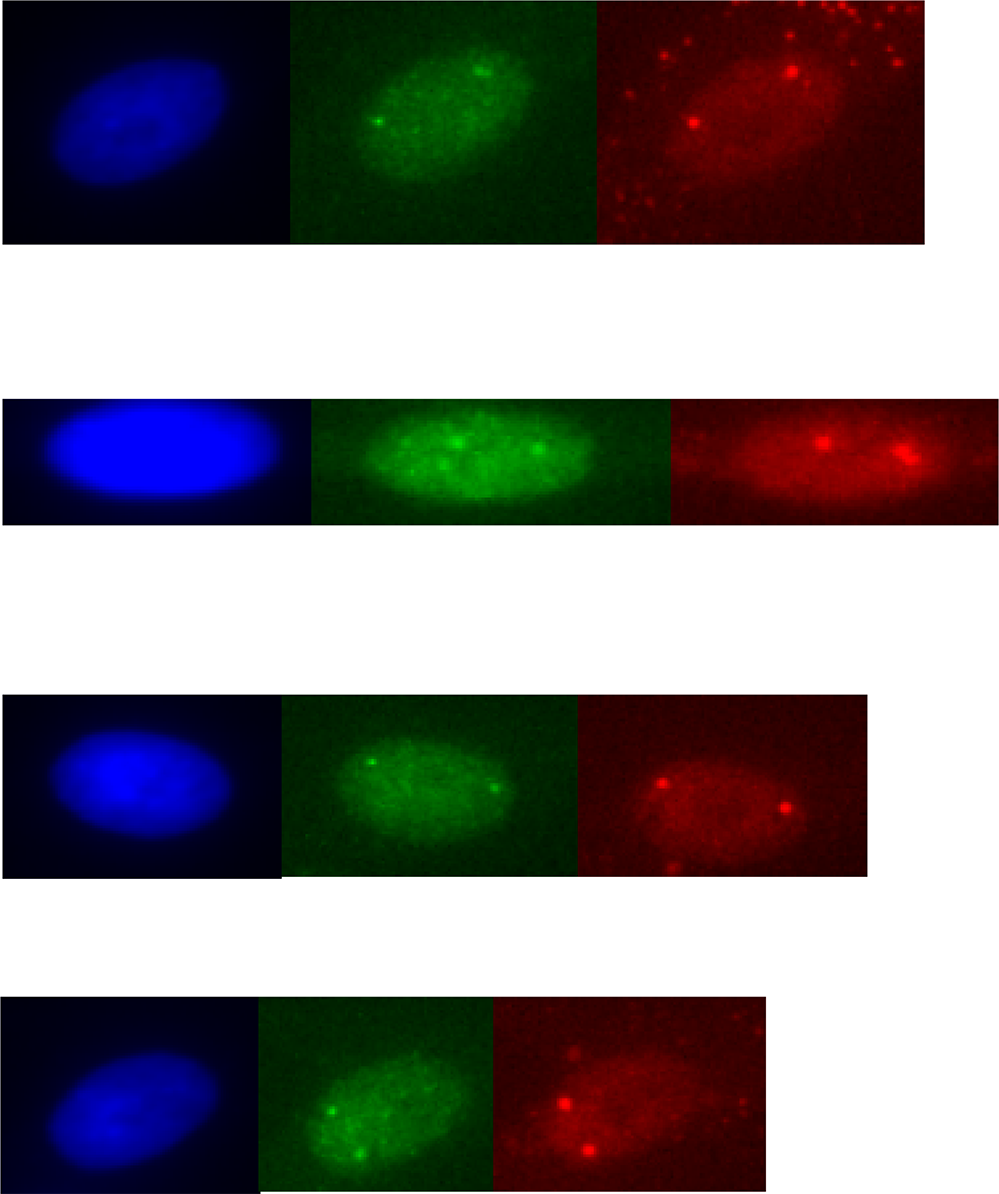

Supplement: Supplementary file 1 — Maximum intensity projection (MIP) of representative examples with the DAPI channel outlining the nucleus boundary and FITC and TRITC channels showing the gene probe and the sub-telomere probe, respectively. (PNG 260 kb). [file 412_2020_747_Fig6_ESM.png]

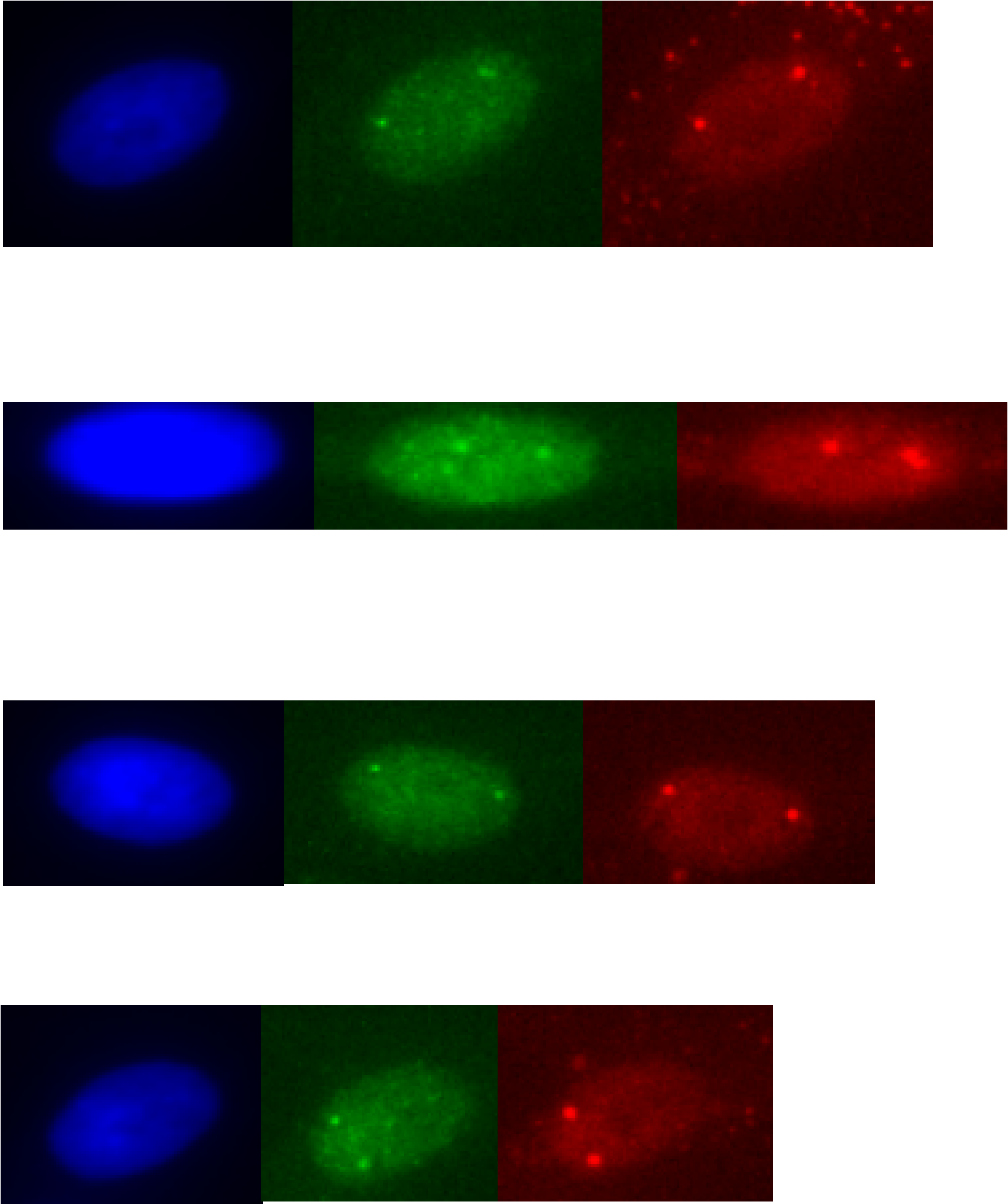

Supplement: Supplementary file 2 — High resolution image (TIF 15486 kb). [file 412_2020_747_MOESM1_ESM.tif]

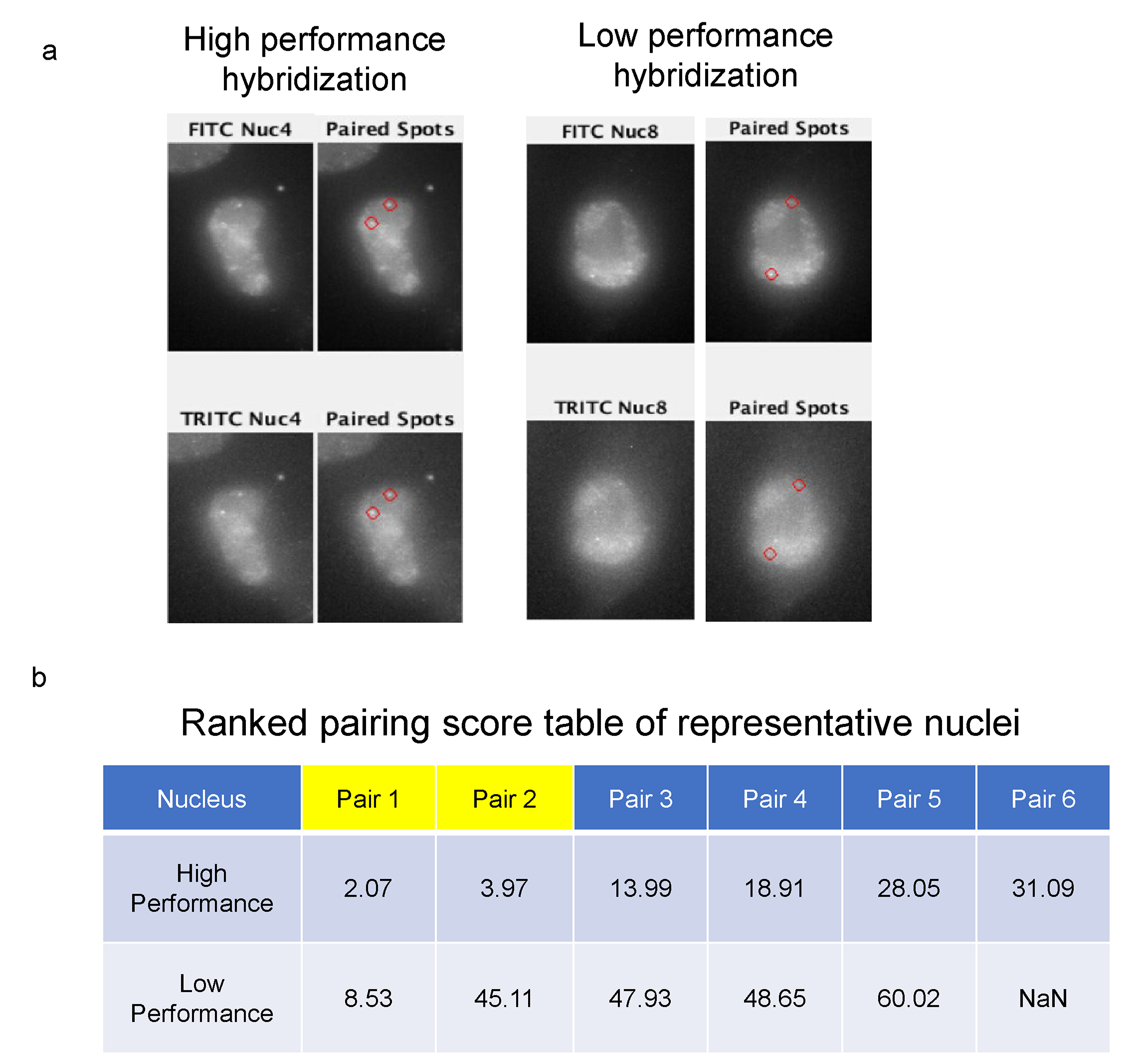

Supplement: Supplementary file 3 — Quality control of spot pairing. (a) Examples of high-performance hybridization and low performance hybridization in spot pairing. Red circles indicate the positions of top 2 pairs in each nucleus. (b) Ranked pairing scores of selected nuclei. With high performance hybridization, the top two candidates have significantly lower score compared to the other scores. With low performance hybridization the scores of the two candidates are not clearly different from the other scores. (PNG 604 kb). [file 412_2020_747_Fig7_ESM.png]

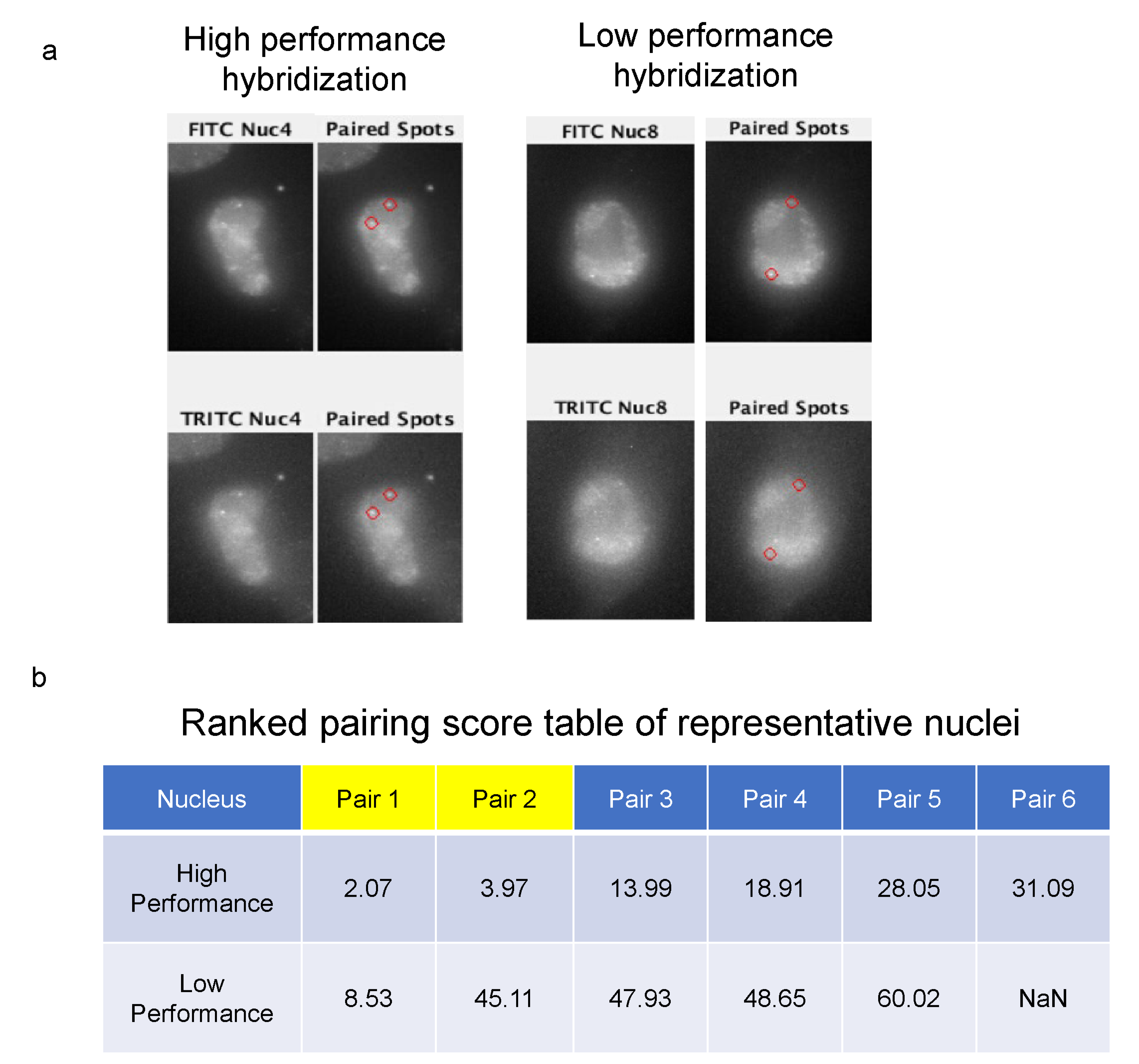

Supplement: Supplementary file 4 — High resolution image (TIF 63431 kb). [file 412_2020_747_MOESM2_ESM.tif]

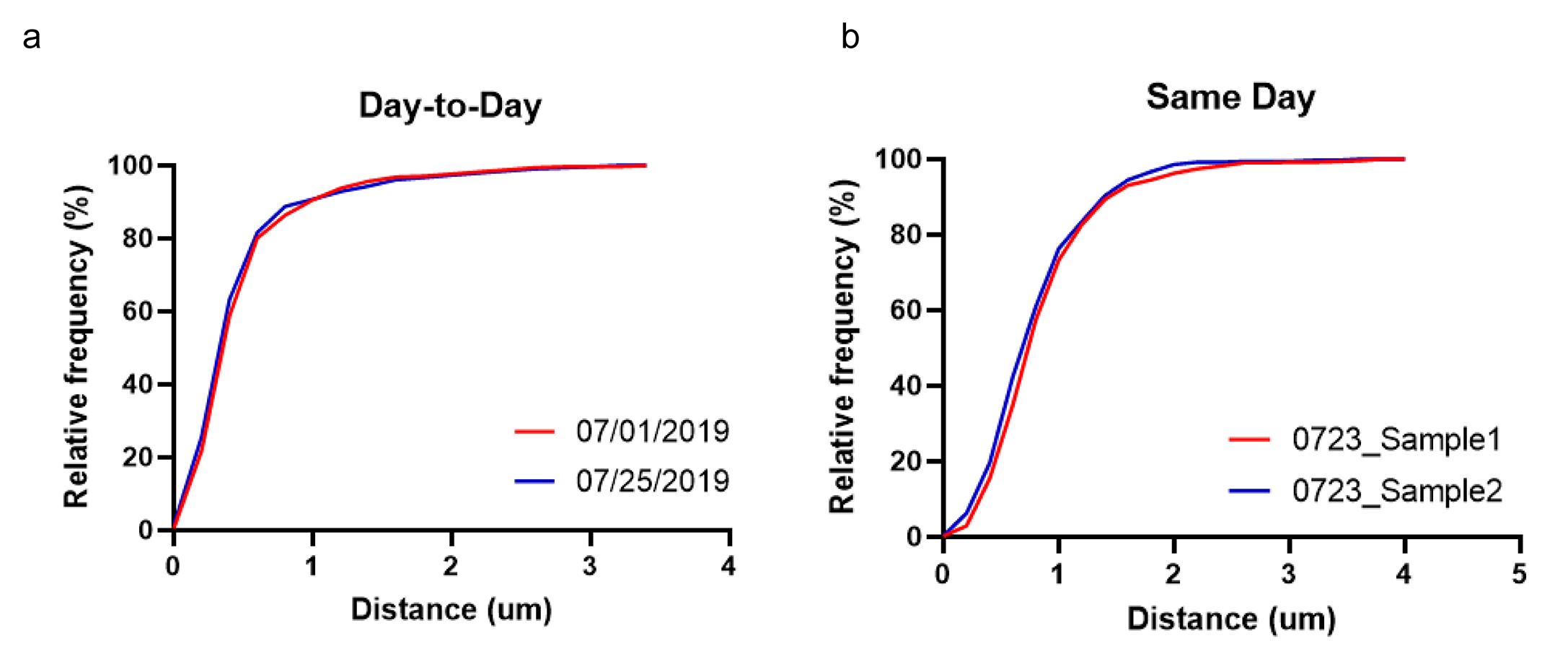

Supplement: Supplementary file 5 — hTERT immortalized cells analyzed between (a) two different days or (b) on the same day, but on different glass slides. No significant difference was found between the distributions of these data groups (p-values of 0.1 and 0.08 for the day-to-day and same-day comparisons, respectively). (PNG 236 kb). [file 412_2020_747_Fig8_ESM.png]

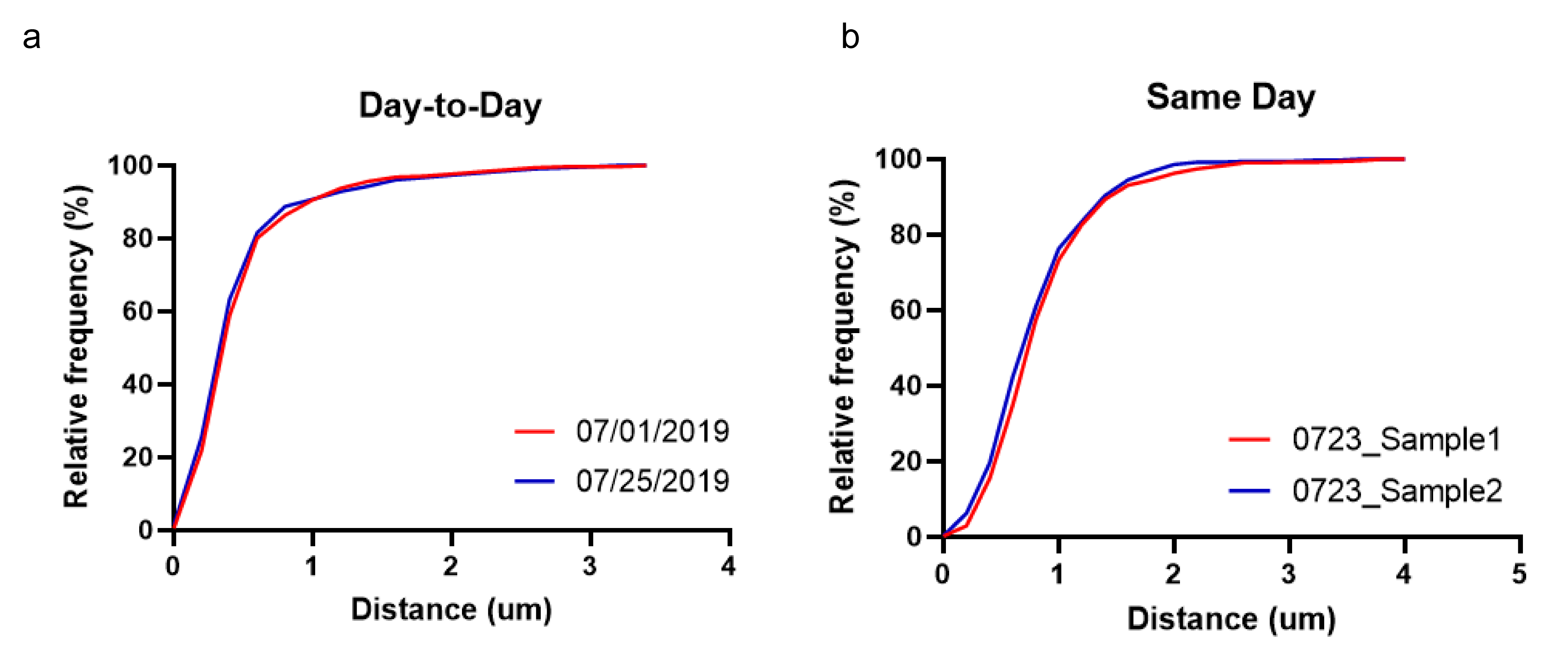

Supplement: Supplementary file 6 — High resolution image (TIF 24439 kb). [file 412_2020_747_MOESM3_ESM.tif]

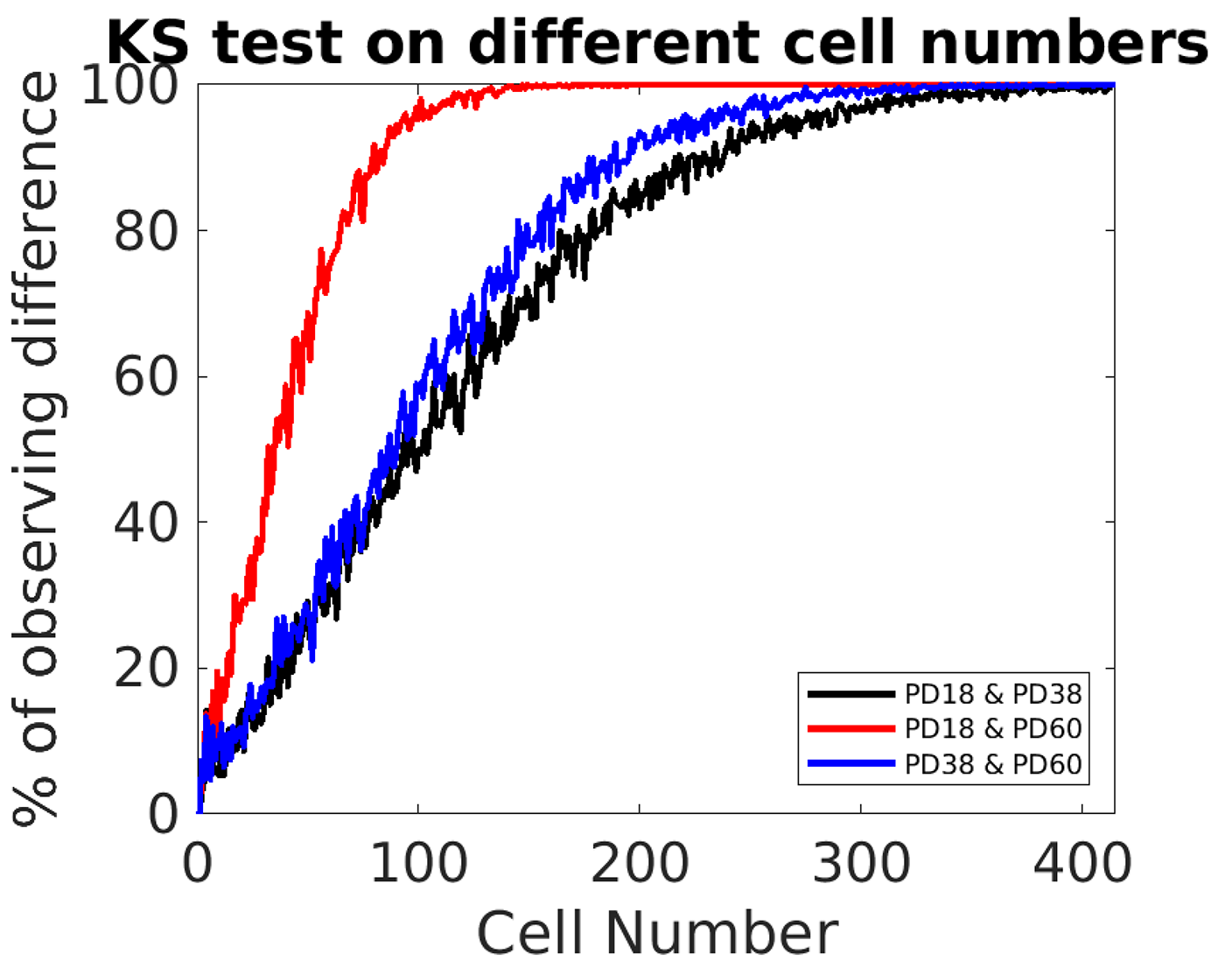

Supplement: Supplementary file 7 — Large cell number quantification is required for statistical robustness. Large cell number is required for quantification. Designated number of cells were randomly selected from the PD18, PD38 and PD60 cell pools using bootstrap. Only with more than 150 cells can the difference between PD18 and PD60 be reproducibly observed. More cells are required if the two samples have closer PDs. (PNG 220 kb). [file 412_2020_747_Fig9_ESM.png]

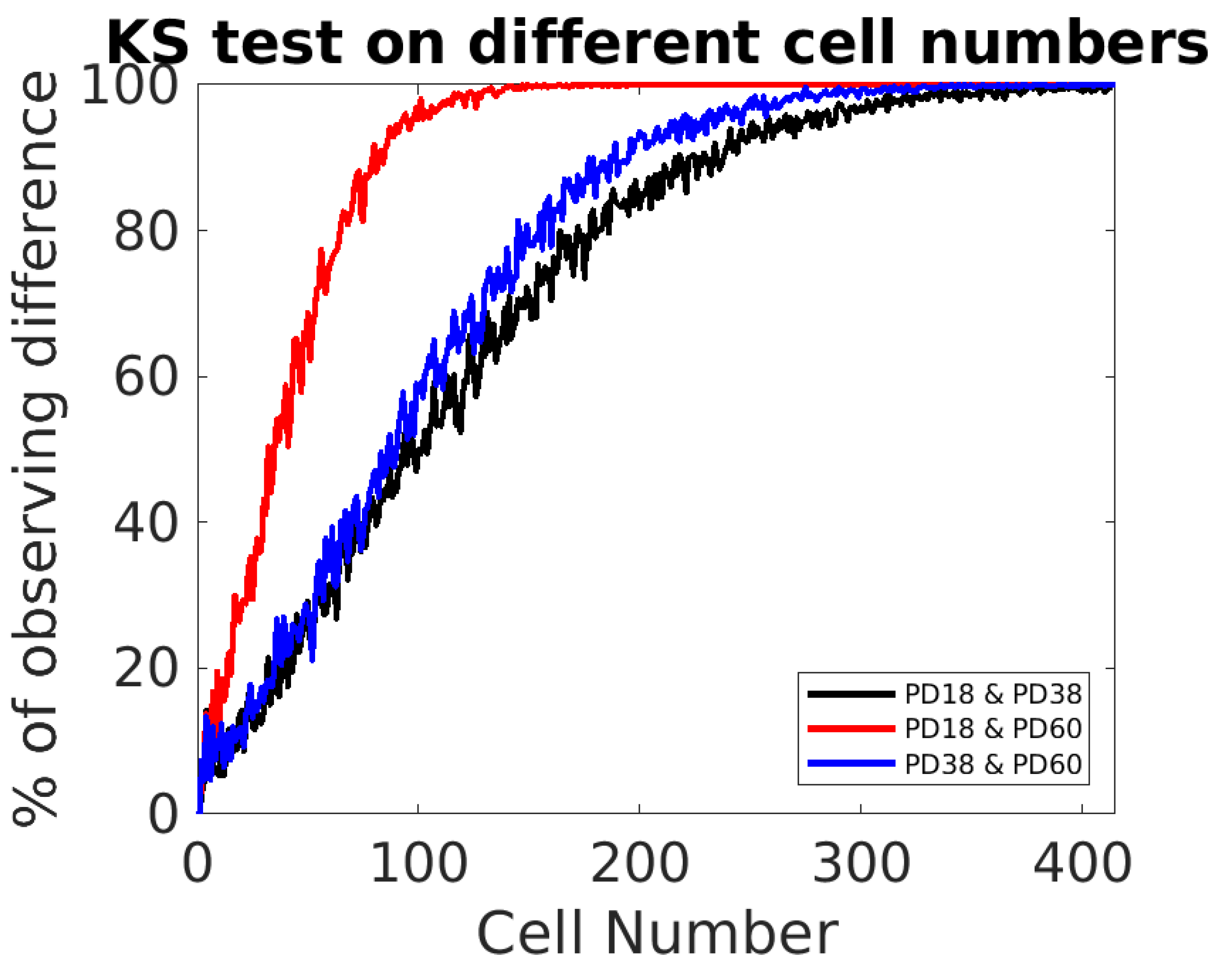

Supplement: Supplementary file 8 — High resolution image (TIF 19503 kb). [file 412_2020_747_MOESM4_ESM.tif]

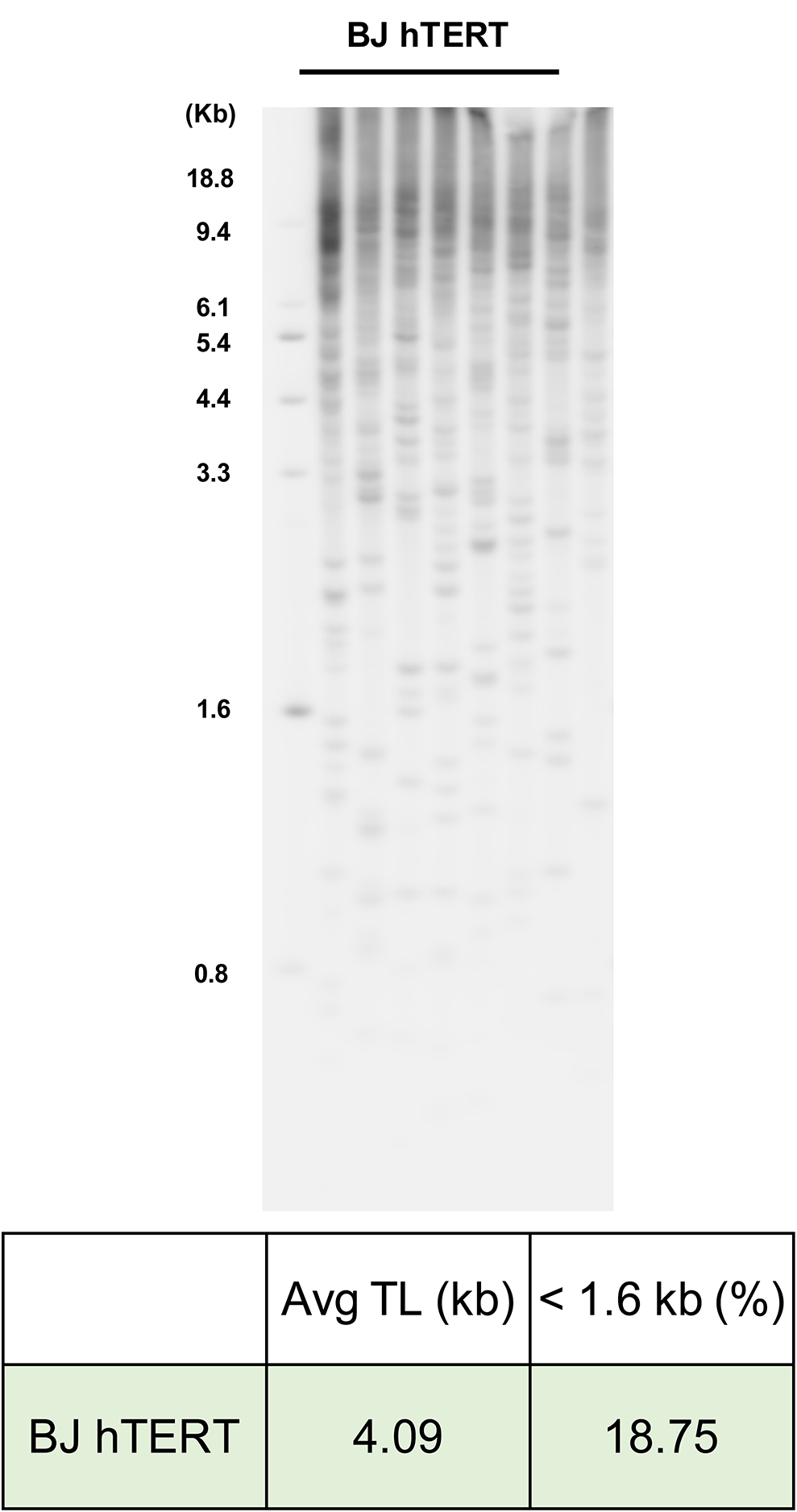

Supplement: Supplementary file 9 — TeSLA of TERT immortalized BJ cells shows the re-elongated telomere length. Telomere length distribution revealed by Telomere Shortest Length Assay (TeSLA) for hTERT immortalized old human fibroblasts (BJ cells). Its telomere length was re-elongated due to the function of telomerase after cultivation. (PNG 148 kb). [file 412_2020_747_Fig10_ESM.png]

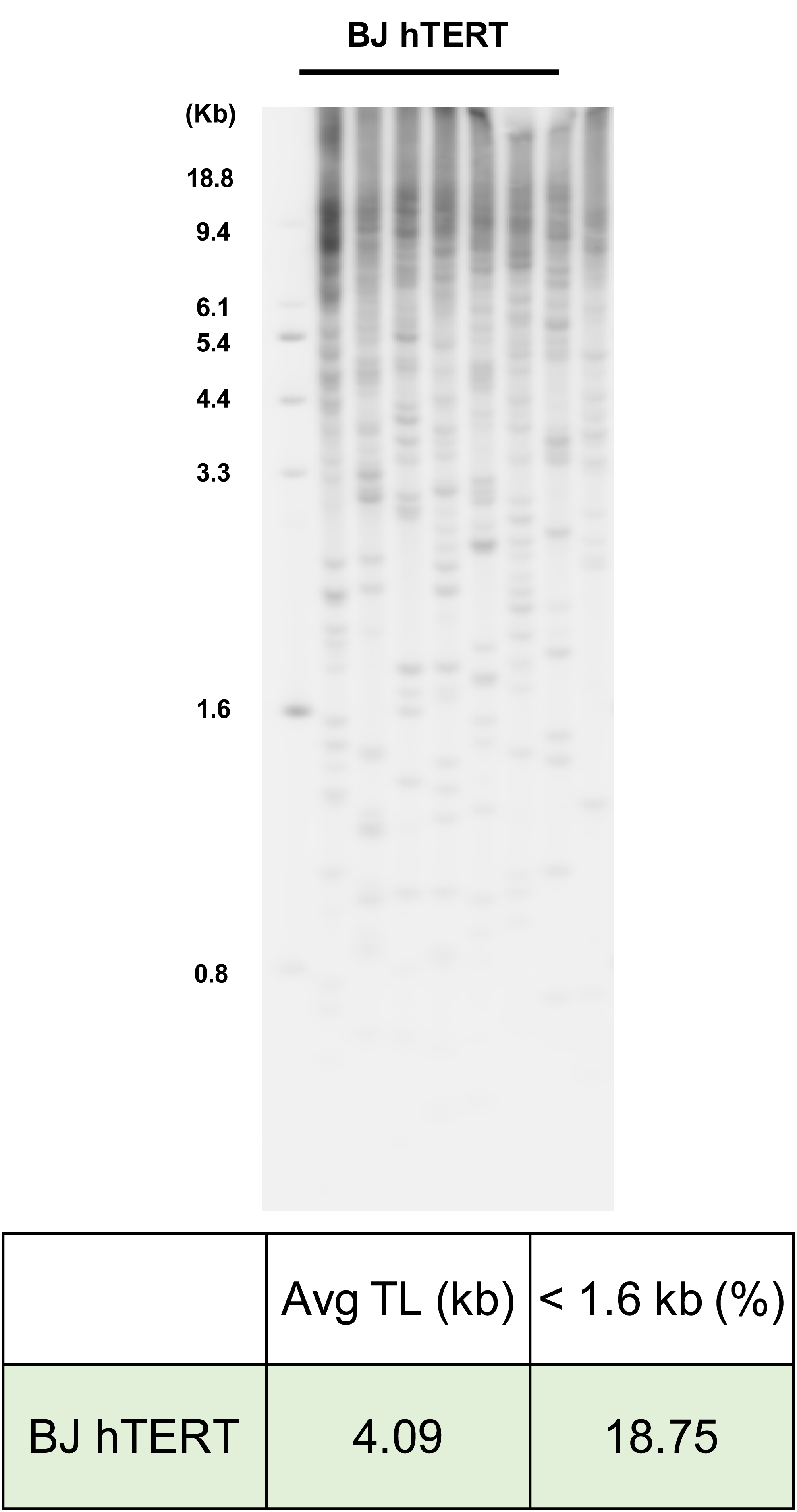

Supplement: Supplementary file 10 — High resolution image (TIF 24404 kb). [file 412_2020_747_MOESM5_ESM.tif]
